# Methylmercury-induced DNA methylation—From epidemiological observations to experimental evidence

**DOI:** 10.3389/fgene.2022.993387

**Published:** 2022-09-13

**Authors:** Andrea Cediel-Ulloa, Ximiao Yu, Maria Hinojosa, Ylva Johansson, Anna Forsby, Karin Broberg, Joëlle Rüegg

**Affiliations:** ^1^ Department of Organismal Biology, Uppsala University, Uppsala, Sweden; ^2^ Department of Biochemistry and Biophysics, Stockholm University, Stockholm, Sweden; ^3^ Institute of Environmental Medicine, Karolinska Institutet, Stockholm, Sweden

**Keywords:** methyl mercury (MeHg), neurodevelopment, epigenome wide association study, DNA methylation, SH-SY5Y cell line

## Abstract

Methylmercury (MeHg) is a developmental neurotoxicant, and one potential mechanism of MeHg toxicity is epigenetic dysregulation. In a recent meta-analysis of epigenome-wide association studies (EWAS), associations between prenatal MeHg exposure and DNA methylation at several genomic sites were identified in blood from newborns and children. While EWASs reveal human-relevant associations, experimental studies are required to validate the relationship between exposure and DNA methylation changes, and to assess if such changes have implications for gene expression. Herein, we studied DNA methylation and gene expression of five of the top genes identified in the EWAS meta-analysis, MED31, MRPL19, GGH, GRK1, and LYSMD3, upon MeHg exposure in human SH-SY5Y cells exposed to 8 or 40 nM of MeHg during differentiation, using bisulfite-pyrosequencing and qPCR, respectively. The concentrations were selected to cover the range of MeHg concentrations in cord blood (2–8.5 μg/L) observed in the cohorts included in the EWAS. Exposure to MeHg increased DNA methylation at MED31, a transcriptional regulator essential for fetal development. The results were in concordance with the epidemiological findings where more MED31 methylation was associated with higher concentrations of MeHg. Additionally, we found a non-significant decrease in DNA methylation at GGH, which corresponds to the direction of change observed in the EWAS, and a significant correlation of GGH methylation with its expression. In conclusion, this study corroborates some of the EWAS findings and puts forward candidate genes involved in MeHg’s effects on the developing brain, thus highlighting the value of experimental validation of epidemiological association studies.

## Introduction

Methylmercury (MeHg), an organic form of mercury, is a developmental neurotoxicant. Epidemiological studies have reported severe neurodevelopmental impairment in population exposed to high concentrations of MeHg (Bakir et al., 1973; Harada, 1995; Grandjean and Landrigan, 2014), and experimental data show that exposure to low concentrations of MeHg interferes with important neurodevelopmental processes such as neural proliferation, neuronal migration and neurite outgrowth (Parran et al., 2001; Tamm et al., 2006; Guo et al., 2013; Fujimura and Usuki, 2015; Attoff et al., 2017).

Exposure to MeHg in the general population occurs predominantly by ingestion of fish and sea food (Sheehan et al., 2014; Nogara et al., 2019). Once absorbed, MeHg mainly binds to hemoglobin and is distributed to different tissues throughout the body (Pan et al., 2022). During pregnancy, MeHg can cross the placenta and the blood-brain barrier (Kajiwara et al., 1996), making the developing brain a sensitive target. There is uncertainty whether MeHg exposure from consumption of fish with background MeHg levels causes neurodevelopmental effects (Grandjean et al., 1997; Debes et al., 2006; Llop et al., 2012; Strain et al., 2015; Vejrup et al., 2016; van Wijngaarden et al., 2017; Barbone et al., 2019; Ke et al., 2021). However, epidemiological studies on epigenetic and other biomarkers of effects have uncovered potential toxicity from MeHg exposure early in life (Al-Saleh et al., 2016; Cardenas et al., 2017; Xu et al., 2019).

One of the mechanisms by which MeHg may induce developmental neurotoxicity is through epigenetic modifications (Pan et al., 2022). In fact, vital neurodevelopmental processes such as neurogenesis, astrogliogenesis, and neuronal differentiation and migration are dependent on epigenetic regulation (Gapp et al., 2014). DNA methylation, the addition of a methyl group at the fifth carbon of a pyrimidine base, is a major type of epigenetic modification with implications on the programming of different cells, including those in the brain (Jang et al., 2017). Moreover, DNA methylation can be affected by chemical exposure (Pan et al., 2022). For example, DNA hypomethylation in neural stem cell has been reported after exposure to 2.5 and 5 nM of MeHg (Bose et al., 2012). Several epidemiological studies have also reported associations between exposure to MeHg and altered DNA methylation at specific loci (Bakulski et al., 2015; Appleton et al., 2017; Cediel Ulloa et al., 2021; Nishizawa-Jotaki et al., 2021). A recent large epigenome-wide association study (EWAS) by Lozano et al. (2022) demonstrated associations between prenatal exposure to MeHg and altered DNA methylation at several loci in blood from newborns (n = 1,462) and children (n = 883), further supporting epidemiological associations between developmental exposure to MeHg and altered DNA methylation. Nonetheless, associations in human data can be confounded by other factors and implications on gene expression and biological functions remain unknown.

In order to corroborate the findings from Lozano et al. and to elucidate whether these could be relevant for the developing brain (the main target of MeHg toxicity), we studied DNA methylation changes in an *in vitro* model previously used for the study of developmental neurotoxicity, the SH-SY5Y cell-line which displays characteristics of dopaminergic neurons (Presgraves et al., 2004; Lopes et al., 2010). We selected the five top differentially methylated genes from Lozano et al. (*MED31*, *MRPL19*, *GGH*, *GRK1*, and *LYSMD3*), and analyzed DNA methylation changes induced by exposure to MeHg during cellular differentiation. *MED31*, *GGH* and *GRK1* were selected based on the fact that they were the only genes whose DNA methylation consistently associated with MeHg exposure in all cohorts included in the Lozano meta-analysis. Additionally, *MRPL19* and *LYSMD3,* whose DNA methylation associated with MeHg in some studies, were selected since their expression has been reported to change upon exposure to MeHg in human embryonic stem cell-derived neural ectodermal progenitor cells (Waldmann et al., 2017). Moreover, in order to investigate whether the methylation changes could influence gene expression during neurodevelopment, we analyzed the expression of the selected genes.

## Methods

### Human SH-SY5H cell culture

SH-SY5Y cells were cultured in accordance with an established protocol (Attoff et al., 2016), routinely checked for *mycoplasma* contamination and used between passages 60–65. Cells were maintained in Minimum Essential Media containing 10% FBS, 1% Non-Essential Amino Acids, 2 mM L-glutamine, and 1% Penicillin-Streptomycin (all reagents from Life Technologies). Cell cultures were sub-cultivated once every week, cells were seeded at a density of 27,000 cells/cm^2^ in 75 cm^2^ cell culture flasks, and kept in an incubator at 37°C and 5% of CO_2_. For the experiments, SH-SY5Y cells were seeded in 60 mm × 15 mm dishes at a density of 12,500 cells/cm^2^. One day after seeding, the routine culture medium was removed and replaced with differentiation medium composed of DMEM:F12 supplemented with 100 IU/ml penicillin and 100 μg/ml streptomycin, 2 mM L-glutamine, 1% N2 supplements and 1 µM retinoic acid (Merck), dissolved in ethanol (final concentration 0.1%). The exposure to MeHg took place over 6 days while the cells were differentiating. For this, the exposure was started the first day of differentiation and 50% of the differentiation medium was replaced with fresh differentiation medium containing 1X MeHg 3 days later.

At the end of the differentiation and exposure, the media from the SH-SY5Y cells was removed and the cells were washed once with PBS (Gibco/Life technologies). To detach the cells, 1 ml of TrypLE (Gibco/Life technologies) were added to each well and left to incubate for 5 min at room temperature. The cells were then re-suspended in 2 ml of routine culture media, transferred to 15 ml tubes and centrifuged for 5 min at 300 g. The supernatant was then removed and the cells were kept at −80°C until the RNA and DNA extraction was carried out.

### Exposure to MeHg

MeHgCl (Alfa Aesar, CAS 115-09-3) was dissolved in DMSO in polypropylene Eppendorf tubes and stock solutions of 1 mM were stored at −20°C until use. In order to cover the range of MeHg concentrations (2–8.5 μg/L) in cord blood observed in Lozano et al. (2022), SH-SY5Y cells were exposed to 8 and 40 nM of MeHg. These concentrations were calculated based on a molecular weight of 213.63 g/ml for MeHgCl and are equivalent to 1.7 and 8.5 μg/L respectively. Control cells were exposed to 0.1% DMSO. Effects on cell viability were assessed by visual examination at the end of the exposure.

### RNA and DNA extraction

For extraction of RNA and DNA, the AllPrep DNA/RNA Mini Kit (Qiagen) was used following the manufacturer’s instructions. The concentrations of the extracted RNA and DNA were quantified with a plate reader (Tecan Spark, Zürich, Switzerland) or with a Nanophotometer P-class (IMPLEN GmbH), and the samples were stored at −80°C until further processing.

### Gene expression

Extracted RNA was diluted to a concentration of 1,000 ng in 20 µl RNase free water, and cDNA was synthetized with iScript Synthesis Kit (BioRad), following the manufacturer’s instructions. Upon cDNA synthesis, the samples were diluted in 60 µl of RNAse free water and stored at −20°C until processing. The quantitative polymerase chain reaction (RT-qPCR) was performed using 4 µl of cDNA (4 ng), 5 µl SsoAdv supermix (BioRad), primers, and RNase free water. Amplification was carried out with a CFX384 Touch Real-Time PCR Detection System (Bio-Rad, Hercules, California) using a program of 95°C for 2 min; 95°C, 5 s and 60°C, 30 s with 40 cycles and melt curve analysis at 65–95°C for 5 s. The primers were purchased as PrimePCR assays from Bio-Rad and the assay information is provided in the (Supplementary Table S1). Data were analyzed with the CFX Maestro software (Bio-Rad, version 1.1), the CT values of SH-SY5Y samples were normalized against three reference genes (*RPL19*, *TBP*, and *POLR2B*). The relative gene expression was calculated using the 2ˆ–delta delta CT (2^^-ΔΔCT^) method (Livak and Schmittgen, 2001), and the analyzed data are presented as Log2-fold change in expression of each gene at different MeHg concentrations.

### DNA methylation

Bisulfite treatment was performed on 200 ng DNA using the EZ DNA Methylation-Gold Kit (Zymo Research, Irvine, CA, United States) following the manufacturer’s instructions. The bisulfite treated DNA was stored at −20°C and used for pyrosequencing. Gene fragments were amplified with a T100 Thermal Cycler (Bio-Rad, Hercules, California). For pyrosequencing, 10 µl of the PCR products were mixed with binding buffer (Qiagen), beads (Cytiva) and ultrapure Milli-Q water. After shaking at 1,400 rpm for 20–25 min, the beads containing immobilized template DNA were captured onto filter probes and run through different buffers. Subsequently, the attached DNA templates were released into the plate containing sequencing primer, and incubated at 80°C for 2 min. Finally, substrate, enzyme, and nucleotides (dNTP; deoxynucleoside triphosphate) from PyroMark Gold Q24 Reagents (Qiagen) were loaded into the reagent cartridge, allowing them to be injected into the plate. The assay conditions used for PCR and pyrosequencing of the analyzed genes are presented in Supplementary Table S2. PyroMark Q24 ID (Qiagen, Hilden, Germany) was used for sequencing and the percentage of DNA methylation was calculated with PyroMark Q24 software (Qiagen PyroMark Q24, v. 5.0).

### Prediction of transcription factor binding sites

The prediction of transcription factor binding to the analyzed sequences was carried out with the University of California-Santa Cruz Genome Browser (http://genome.ucsc.edu/) and the JASPAR2022 TFBS hg19 track (Castro-Mondragon et al., 2022). This track permits the visualization of genome-wide transcription factor binding sites (TFBS) available in the JASPAR database CORE collection. The predicted TFBS are reported in Supplementary Table S3.

### Statistics

Comparison between controls and treatment was carried out with One-way Analysis of variance (ANOVA) with Dunnett’s post-hoc test. Bonferroni correction was carried out to adjust for multiple comparisons, unadjusted and adjusted *p*-values are reported in Supplementary Table S4. Correlation analyses between DNA methylation and gene expression were performed with Spearman correlations. Statistical significance was considered for *p*-values <0.05. All the statistical analyses were executed with RStudio Version 1.2.5033 (RStudio, 2019).

## Results

**FIGURE 1:**
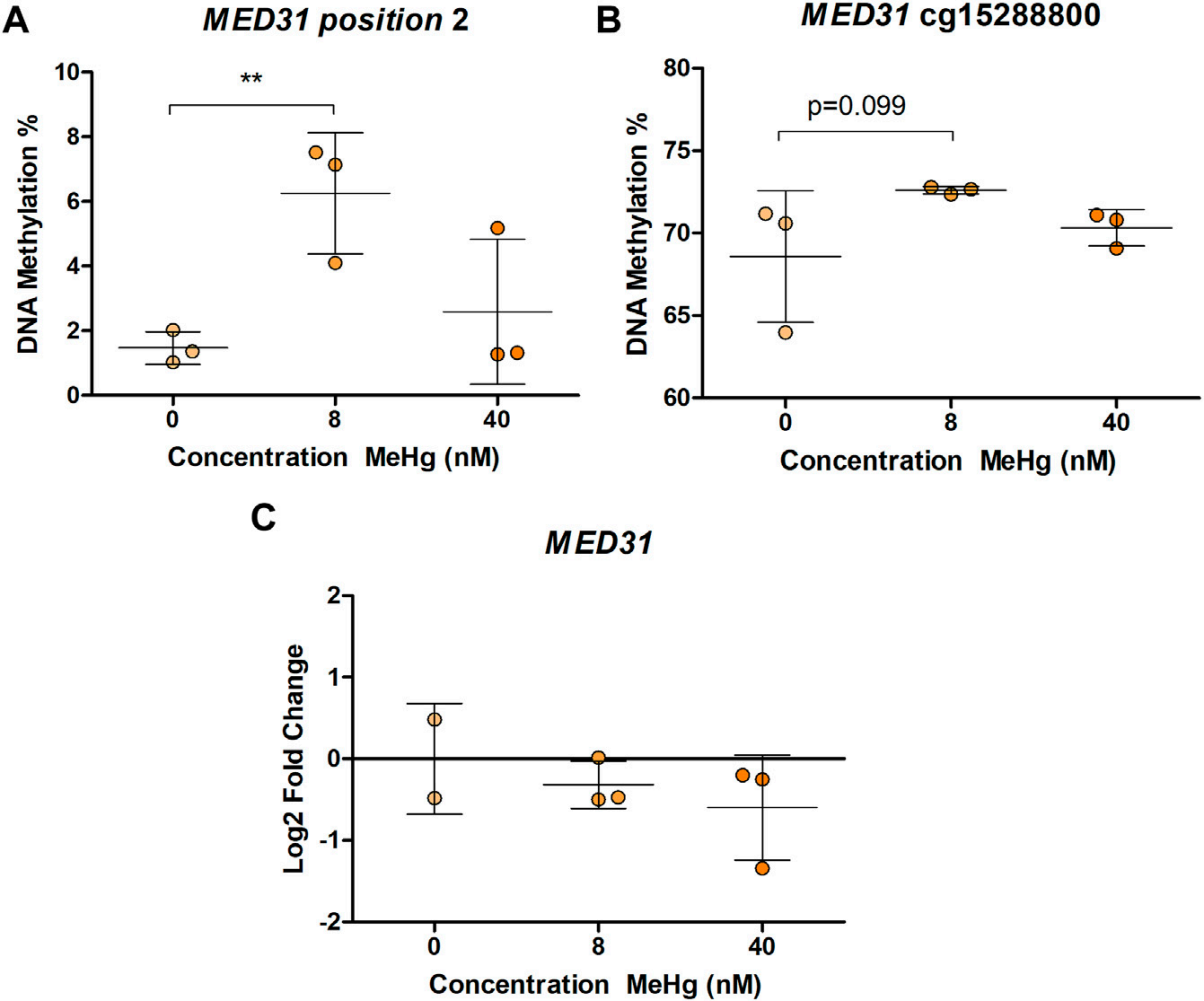
*MED31* DNA methylation and gene expression in MeHg-exposed SH-SY5Y cells; **(A)** DNA methylation levels at position chr17: 6,555,443 located 3 bp from cg24184221; **(B)** DNA methylation levels at position chr17: 6,555,742; **(C)** *MED31* expression levels. The results are presented as the mean ± SD of three independent biological replicates and were analyzed with One-way Analysis of variance (ANOVA) with Dunnett’s post-hoc test. Statistical significance was considered when the *p*-value was below 0.05.

**FIGURE 2:**
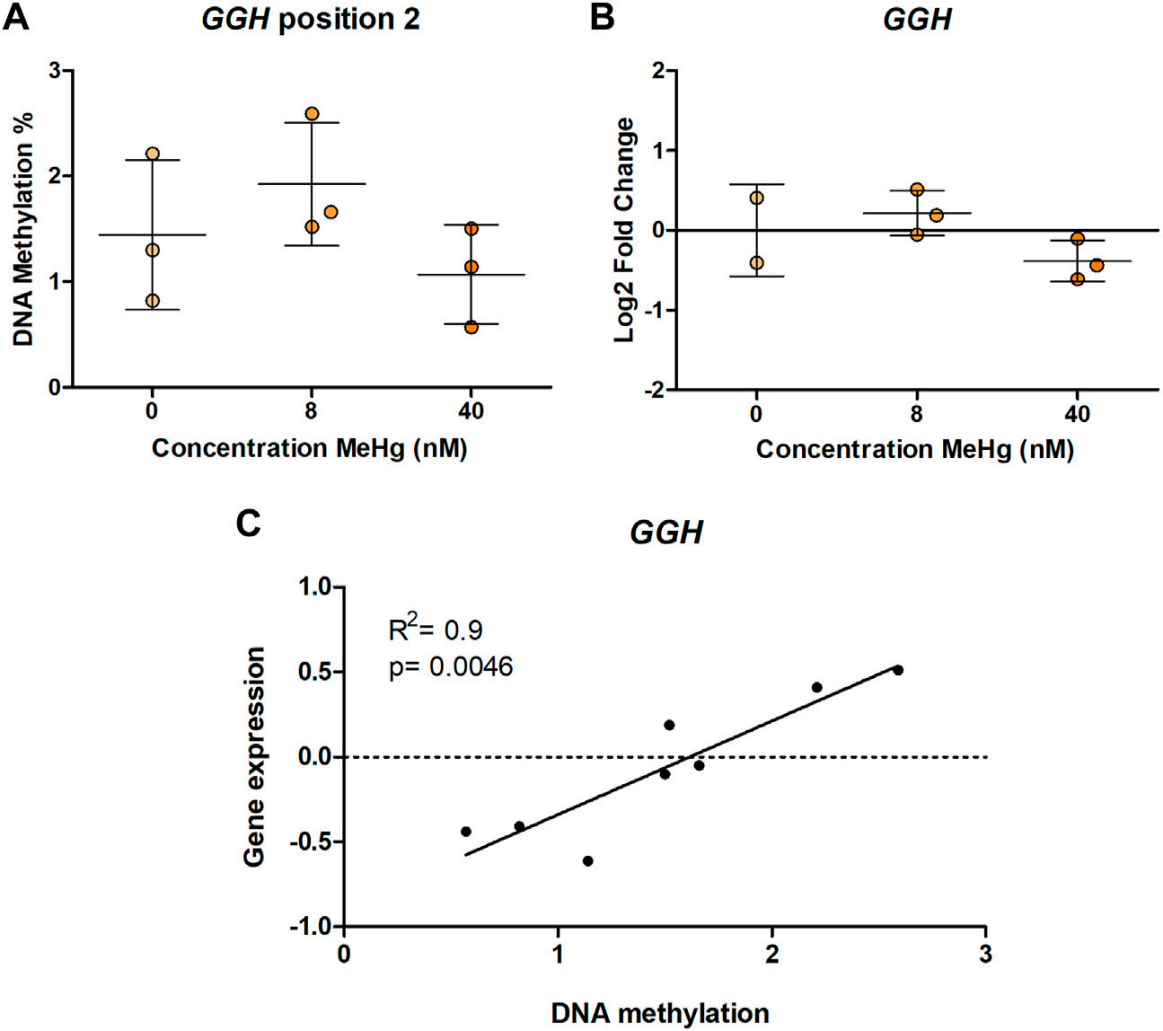
*GGH* DNA methylation and gene expression in MeHg-exposed SH-SY5Y cells; **(A)** DNA methylation levels, **(B)** gene expression, and **(C)** Spearman correlation between gene expression and DNA methylation. The results are presented as the mean ± SD of three independent biological replicates and were analyzed with One-way Analysis of variance (ANOVA) with Dunnett’s post-hoc test. Statistical significance was considered when the *p*-value was below 0.05.

In human SH-SY5Y cells, exposure to MeHg 8 nM led to a significant increase (*p* = 0.00175, adjusted *p* = 0.0385) in DNA methylation of one of the CpG sites in *MED31* (Figure 1A). This CpG is located 3 base pairs (bp) from cg24184221, which was the one identified in Lozano et al. (2022) to be more methylated with higher exposure to MeHg. A similar, but non-significant (*p* = 0.0988, adjusted *p* = 1) change was found on DNA methylation of cg15288800 (Figure 1B), another CpG in the *MED31* identified by Lozano et al. (2022) to be more methylated with higher exposure to MeHg. Exposure to 40 nM MeHg did not produce significant changes on *MED31* methylation nor was *MED31* expression significantly altered, although a trend towards downregulation was observed (Figure 1C). MeHg exposure did not reveal any statistically significant changes (*p* > 0.05) on DNA methylation and gene expression of *MRPL19*, *GGH*, *GRK1*, and *LYSMD3* (Figures 2A,B and Supplementary Figures S1, S2), nonetheless, trends were observed that were in accordance with the EWAS findings.

Although we did not evidence gene expression changes on any of the studied genes, we used the data to clarify if DNA methylation at the studied regions is correlated with mRNA expression, which would indicate a functional role of the DNA methylation changes observed in humans. Our results showed a positive correlation between DNA methylation and gene expression of *GGH* CpG2 (r_S_ = 0.9, *p* = 0.0046) (Figure 2C). No other statistically significant correlations were observed (Supplementary Table S5).

## Discussion

The aim of this study was to corroborate and address functionality of associations between prenatal MeHg exposure and DNA methylation levels identified in an EWAS with experimental data. We were able to corroborate findings for *MED31* at an adjacent CpG site of the one reported in the EWAS meta-analysis, even after adjusting for multiple comparisons. *MED31* encodes for the mediator complex subunit 31 and is expressed in a variety of mouse fetal tissues with the highest expression in the developing brain (Risley et al., 2010). MED31 is part of the mediator complex transcriptional activator responsible for mediating polymerase II promoter-enhancer interactions, and hence functioning as a major transcriptional regulator (Richter et al., 2022). Due to its function, the mediator complex is essential for fetal development, including neurodevelopment. For example, in neural stem cells (NSC), the mediator complex regulates expression of neurogenic transcription factors and genes linked to NSC identity (Quevedo et al., 2019). While *MED31* expression was not significantly changed by MeHg in the differentiated SH-SY5Y cells, the observed DNA methylation alteration could still be relevant for gene expression in other cell types. Indeed 14 transcription factors (HIF1A, HES5, HES7, MYC, MYCN, MXI1, BHLHE40, CLOCK, HES1, HEY1, HEY2, MAX, MNT, and NPAS2, Supplementary Table S3) are predicted to bind to the analyzed region. Binding of some of these transcription factors is affected by DNA methylation, e.g., HIF1A, MYCN, and MAX whose binding is inhibited by DNA methylation (Cusack et al., 2020; D’Anna et al., 2020; Perini et al., 2005; Weinmann and Farnham, 2002) or NPAS2 that preferentially binds to methylated CpGs (Zhu et al., 2016). Thus, changes in *MED31* methylation could be functionally implicated in the neurodevelopmental effects of MeHg.

Moreover, we identified correlations between DNA methylation and gene expression for *GGH*, implicating that altered DNA methylation at these positions influences gene expression. *GGH* encodes Gamma-glutamyl hydrolase, an enzyme involved in folate metabolism (Gibson et al., 2011). *GGH* is widely expressed, however, its expression is particularly high in dopaminergic neurons in the substantia nigra (Licker et al., 2014), which is interesting considering that SH-SY5Y are a model of dopaminergic cell differentiation. We found a positive correlation between DNA methylation and gene expression in SH-SY5Y, indicating increased gene expression with higher DNA methylation. This correlation was found at a CpG site located 1 bp away from GGH cg02212000. As this CpG site is not covered by the Illumina EPIC and 450K arrays which the EWAS was based on, we do not have information on potential associations between its methylation pattern and developmental exposure to MeHg in humans. Transcription factor binding site analysis predicted binding of transcription factors MGA, ATF3, PAX2, HIFLA, GMEB2, and FOXO4 to the region (Supplementary Table S3), out of which Atf3 acts as a transcriptional repressor. DNA binding of Atf3 has been reported to be impaired by DNA methylation (Zhang et al., 2021). Hence, the observed decrease in *GGH* DNA methylation in the EWAS (Lozano et al., 2022) could potentially lead to increased Atf3 binding and a reduction of gene expression.

No other statistically significant effects and correlations were found in our study, which could imply that MeHg exposure has no direct effect on the other investigated genes. However, we cannot exclude such effects in blood cells (as analyzed in the EWAS) or other cell types and tissues not addressed by the *in vitro* model we have chosen in this study. Additionally, the lack of statistically significant results could be secondary to the small sample size used in this study.

In conclusion, our study highlights the value of experimental validation of epidemiological association studies and suggests a role for *MED31*-controlled processes in the developmental neurotoxicity of MeHg.

## Data Availability

The datasets presented in this study can be found in online repositories. The link to the data can be found below: https://github.com/AndreaCediel/METHYLMERCURY-INDUCED-DNA-METHYLATION-FROM-EPIDEMIOLOGICAL-OBSERVATIONS-TO-EXPERIMENTAL-EVIDENCE.

## References

[B1] Al-Saleh I. Elkhatib R. Al-Rouqi R. Abduljabbar M. Eltabache C. Al-Rajudi T. (2016). Alterations in biochemical markers due to mercury (Hg) exposure and its influence on infant's neurodevelopment. Int. J. Hyg. Environ. Health 219, 898–914. 10.1016/j.ijheh.2016.07.002 27453562

[B2] Appleton A. A. Jackson B. P. Karagas M. Marsit C. J. (2017). Prenatal exposure to neurotoxic metals is associated with increased placental glucocorticoid receptor DNA methylation. Epigenetics 12, 607–615. 10.1080/15592294.2017.1320637 28548590 PMC5687337

[B3] Attoff K. Gliga A. Lundqvist J. Norinder U. Forsby A. (2017). Whole genome microarray analysis of neural progenitor C17.2 cells during differentiation and validation of 30 neural mRNA biomarkers for estimation of developmental neurotoxicity. PLoS One 12, e0190066. 10.1371/journal.pone.0190066 29261810 PMC5738075

[B4] Attoff K. Kertika D. Lundqvist J. OredSSon S. Forsby A. (2016). Acrylamide affects proliferation and differentiation of the neural progenitor cell line C17.2 and the neuroblastoma cell line SH-SY5Y. Toxicol. Vitro 35, 100–111. 10.1016/j.tiv.2016.05.014 27241584

[B5] Bakir F. Damluji S. F. Amin-Zaki L. MurtadhaM. KhAlidi A. al-Rawi N. Y. (1973). Methylmercury poisoning in Iraq. Science 181, 230–241. 10.1126/science.181.4096.230 4719063

[B6] Bakulski K. M. Lee H. Feinberg J. I. Wells E. M. Brown S. Herbstman J. B. (2015). Prenatal mercury concentration is associated with changes in DNA methylation at TCEANC2 in newborns. Int. J. Epidemiol. 44, 1249–1262. 10.1093/ije/dyv032 25906783 PMC4588863

[B7] Barbone F. Rosolen V. Mariuz M. Parpinel M. Casetta A. Sammartano F. (2019). Prenatal mercury exposure and child neurodevelopment outcomes at 18 months: Results from the mediterranean PHIME cohort. Int. J. Hyg. Environ. Health 222, 9–21. 10.1016/j.ijheh.2018.07.011 30057028

[B8] Bose R. Onishchenko N. Edoff K. Janson Lang A. M. Ceccatelli S. (2012). Inherited effects of low-dose exposure to methylmercury in neural stem cells. Toxicol. Sci. 130, 383–390. 10.1093/toxsci/kfs257 22918959

[B9] Cardenas A. Rifas-Shiman S. L. Agha G. Hivert M. F. Litonjua A. A. DeMeo D. L. (2017). Persistent DNA methylation changes associated with prenatal mercury exposure and cognitive performance during childhood. Sci. Rep. 7, 288–313. 10.1038/s41598-017-00384-5 28325913 PMC5428306

[B10] Castro-Mondragon J. A. Riudavets-Puig R. Rauluseviciute I. Lemma R. B. Turchi L. Blanc-Mathieu R. (2022). Jaspar 2022: The 9th release of the open-access database of transcription factor binding profiles. Nucleic Acids Res. 50, D165–d173. 10.1093/nar/gkab1113 34850907 PMC8728201

[B11] Cediel Ulloa A. Gliga A. Love T. M. Pineda D. Mruzek D. W. Watson G. E. (2021). Prenatal methylmercury exposure and DNA methylation in seven-year-old children in the Seychelles Child Development Study. Environ. Int. 147, 106321. 10.1016/j.envint.2020.106321 33340986 PMC11849698

[B12] Cusack M. King H. W. Spingardi P. Kessler B. M. Klose R. J. Kriaucionis S. (2020). Distinct contributions of DNA methylation and histone acetylation to the genomic occupancy of transcription factors. Genome Res. 30, 1393–1406. 10.1101/gr.257576.119 32963030 PMC7605266

[B13] D’Anna F. Van Dyck L. Xiong J. Zhao H. Berrens R. V. Qian J. (2020). DNA methylation repels binding of hypoxia-inducible transcription factors to maintain tumor immunotolerance. Genome Biol. 21, 182–236. 10.1186/s13059-020-02087-z 32718321 PMC7384226

[B14] Debes F. Budtz-Jorgensen E. Weihe P. White R. F. Grandjean P. (2006). Impact of prenatal methylmercury exposure on neurobehavioral function at age 14 years. Neurotoxicol. Teratol. 28, 363–375. 10.1016/j.ntt.2006.02.004 16647838 PMC1543702

[B15] Fujimura M. Usuki F. (2015). Low concentrations of methylmercury inhibit neural progenitor cell proliferation associated with up-regulation of glycogen synthase kinase 3β and subsequent degradation of cyclin E in rats. Toxicol. Appl. Pharmacol. 288, 19–25. 10.1016/j.taap.2015.07.006 26184774

[B16] Gapp K. Woldemichael B. T. Bohacek J. Mansuy I. M. (2014). Epigenetic regulation in neurodevelopment and neurodegenerative diseases. Neuroscience 264, 99–111. 10.1016/j.neuroscience.2012.11.040 23256926

[B17] Gibson T. M. Brennan P. Han S. Karami S. Zaridze D. Janout V. (2011). Comprehensive evaluation of one-carbon metabolism pathway gene variants and renal cell cancer risk. PLoS One 6, e26165. 10.1371/journal.pone.0026165 22039442 PMC3198392

[B18] Grandjean P. Landrigan P. J. (2014). Neurobehavioural effects of developmental toxicity. Lancet. Neurol. 13, 330–338. 10.1016/S1474-4422(13)70278-3 24556010 PMC4418502

[B19] Grandjean P. Weihe P. White R. F. DebesF. Araki S. YoKoyama K. (1997). Cognitive deficit in 7-year-old children with prenatal exposure to methylmercury. Neurotoxicol. Teratol. 19, 417–428. 10.1016/s0892-0362(97)00097-4 9392777

[B20] Guo B. Q. Yan C. H. Cai S. Z. Yuan X. B. Shen X. M. (2013). Low level prenatal exposure to methylmercury disrupts neuronal migration in the developing rat cerebral cortex. Toxicology 304, 57–68. 10.1016/j.tox.2012.11.019 23220560

[B21] Harada M. (1995). Minamata disease: Methylmercury poisoning in Japan caused by environmental pollution. Crit. Rev. Toxicol. 25, 1–24. 10.3109/10408449509089885 7734058

[B22] Jang H. S. Shin W. J Jeong Eon Lee J. E Do J. T, (2017). CpG and non-CpG methylation in epigenetic gene regulation and brain function. Genes (Basel). 8 (6), 148. 10.3390/genes8060148 PMC5485512 28545252

[B23] Kajiwara Y. YAsutAke A. Adachi T. Hirayama K. (1996). Methylmercury transport across the placenta via neutral amino acid carrier. Arch. Toxicol. 70, 310–314. 10.1007/s002040050279 8852703

[B24] Ke T. Tinkov A. A. Skalny A. V. Bowman A. B. Rocha J. B. T. Santamaria A. (2021). Developmental exposure to methylmercury and ADHD, a literature review of epigenetic studies. Environ. Epigenet. 7, dvab014. 10.1093/eep/dvab014 34881051 PMC8648069

[B25] Licker V. Turck N. Kovari E. Burkhardt K. Cote M. Surini-Demiri M. (2014). Proteomic analysis of human substantia nigra identifies novel candidates involved in Parkinson's disease pathogenesis. Proteomics 14, 784–794. 10.1002/pmic.201300342 24449343

[B26] Livak K. J. Schmittgen T. D. (2001). Analysis of relative gene expression data using real-time quantitative PCR and the 2(-Delta Delta C(T)) Method. Methods 25, 402–408. 10.1006/meth.2001.1262 11846609

[B27] Llop S. Guxens M. Murcia M. Lertxundi A. Ramon R. Riano I. (2012). Prenatal exposure to mercury and infant neurodevelopment in a multicenter cohort in Spain: Study of potential modifiers. Am. J. Epidemiol. 175, 451–465. 10.1093/aje/kwr328 22287639

[B28] Lopes F. M. Schroder R. da Frota M. L. C. Zanotto-Filho A. Muller C. B. Pires A. S. (2010). Comparison between proliferative and neuron-like SH-SY5Y cells as an *in vitro* model for Parkinson disease studies. Brain Res. 1337, 85–94. 10.1016/j.brainres.2010.03.102 20380819

[B29] Lozano M. Yousefi P. Broberg K. Soler-Blasco R. Miyashita C. Pesce G. (2022). DNA methylation changes associated with prenatal mercury exposure: A meta-analysis of prospective cohort studies from pace consortium. Environ. Res. 204, 112093. 10.1016/j.envres.2021.112093 34562483 PMC10879652

[B30] Nishizawa-Jotaki S. Sakurai K. Eguchi A. Tanabe H. Watanabe M. Mori C. (2021). Association between mercury in cord serum and sex-specific DNA methylation in cord tissues. J. Dev. Orig. Health Dis. 12, 124–131. 10.1017/S2040174420000161 32241331

[B31] Nogara P. A. Oliveira C. S. Schmitz G. L. Piquini P. C. Farina M. Aschner M. (2019). Methylmercury's chemistry: From the environment to the mammalian brain. Biochim. Biophys. Acta. Gen. Subj. 1863, 129284. 10.1016/j.bbagen.2019.01.006 30659885

[B32] Pan J. Li X. Wei Y. Ni L. Xu B. Deng Y. (2022). Advances on the influence of methylmercury exposure during neurodevelopment. Chem. Res. Toxicol. 35, 43–58. 10.1021/acs.chemrestox.1c00255 34989572

[B33] Parran D. K. Mundy W. R. Barone S. (2001). Effects of methylmercury and mercuric chloride on differentiation and cell viability in PC12 cells. Toxicol. Sci. 59, 278–290. 10.1093/toxsci/59.2.278 11158721

[B34] Perini G. Diolaiti D. Porro A. Della Valle G. (2005). *In vivo* transcriptional regulation of N-Myc target genes is controlled by E-box methylation. Proc. Natl. Acad. Sci. U. S. A. 102, 12117–12122. 10.1073/pnas.0409097102 16093321 PMC1184034

[B35] Presgraves S. P. Ahmed T. Borwege S. Joyce J. N. (2004). Terminally differentiated SH-SY5Y cells provide a model system for studying neuroprotective effects of dopamine agonists. Neurotox. Res. 5, 579–598. 10.1007/BF03033178 15111235

[B36] Quevedo M. Meert L. Dekker M. R. Dekkers D. H. W. Brandsma J. H. van den Berg D. L. C. (2019). Publisher Correction: Mediator complex interaction partners organize the transcriptional network that defines neural stem cells. Nat. Commun. 10, 3318–3415. 10.1038/s41467-019-11254-1 31332183 PMC6646368

[B37] Richter W. F. Nayak S. Iwasa J. Taatjes D. J. (2022). The Mediator complex as a master regulator of transcription by RNA polymerase II. Nat. Rev. Mol. Cell Biol. 20:1–18. 10.1038/s41580-022-00498-3 PMC9207880 35725906

[B38] Risley M. D. Clowes C. Yu M. Mitchell K. Hentges K. E. (2010). The Mediator complex protein Med31 is required for embryonic growth and cell proliferation during mammalian development. Dev. Biol. 342, 146–156. 10.1016/j.ydbio.2010.03.019 20347762

[B39] RStudio T. (2019). RStudio: Integrated development for R. Boston, MA: RStudio, Inc. Available at: URL http://www.rstudio.com ,

[B40] Sheehan M. C. Burke T. A. Navas-Acien A. Breysse P. N. McGready J. Fox M. A. (2014). Global methylmercury exposure from seafood consumption and risk of developmental neurotoxicity: A systematic review. Bull. World Health Organ. 92, 254–269f. 10.2471/BLT.12.116152 24700993 PMC3967569

[B41] Strain J. Yeates A. J. van Wijngaarden E. Thurston S. W. Mulhern M. S. McSorley E. M. (2015). Prenatal exposure to methyl mercury from fish consumption and polyunsaturated fatty acids: Associations with child development at 20 mo of age in an observational study in the republic of Seychelles. Am. J. Clin. Nutr. 101, 530–537. 10.3945/ajcn.114.100503 25733638 PMC4340059

[B42] Tamm C. Duckworth J. Hermanson O. Ceccatelli S. (2006). High susceptibility of neural stem cells to methylmercury toxicity: Effects on cell survival and neuronal differentiation. J. Neurochem. 97, 69–78. 10.1111/j.1471-4159.2006.03718.x 16524380

[B43] van Wijngaarden E. Thurston S. W. Myers G. J. Harrington D. Cory-Slechta D. A. Strain J. J. (2017). Methyl mercury exposure and neurodevelopmental outcomes in the Seychelles Child Development Study Main cohort at age 22 and 24 years. Neurotoxicol. Teratol. 59, 35–42. 10.1016/j.ntt.2016.10.011 27989696 PMC5235948

[B44] Vejrup K. Schjolberg S. Knutsen H. K. Kvalem H. E. Brantsæter A. L. Meltzer H. M. (2016). Prenatal methylmercury exposure and language delay at three years of age in the Norwegian Mother and Child Cohort Study. Environ. Int. 92, 63–69. 10.1016/j.envint.2016.03.029 27058928

[B45] Waldmann T. Grinberg M. Konig A. Rempel E. Schildknecht S. Henry M. (2017). Stem cell transcriptome responses and corresponding biomarkers that indicate the transition from adaptive responses to cytotoxicity. Chem. Res. Toxicol. 30, 905–922. 10.1021/acs.chemrestox.6b00259 28001369

[B46] Weinmann A. S. Farnham P. J. (2002). Identification of unknown target genes of human transcription factors using chromatin immunoprecipitation. Methods 26, 37–47. 10.1016/S1046-2023(02)00006-3 12054903

[B47] Xu Y. Wahlberg K. Love T. M. Watson G. E. Yeates A. J. Mulhern M. S. (2019). Associations of blood mercury and fatty acid concentrations with blood mitochondrial DNA copy number in the Seychelles Child Development Nutrition Study. Environ. Int. 124, 278–283. 10.1016/j.envint.2019.01.019 30660840 PMC6405959

[B48] Zhang C. Zhang X. Huang L. Guan Y. Huang X. Tian X. L. (2021). ATF3 drives senescence by reconstructing accessible chromatin profiles. Aging Cell 20, e13315. 10.1111/acel.13315 33539668 PMC7963335

[B49] Zhu H. Wang G. Qian J. (2016). Transcription factors as readers and effectors of DNA methylation. Nat. Rev. Genet. 17, 551–565. 10.1038/nrg.2016.83 27479905 PMC5559737

