# Supplementary material for: Methylmercury-induced DNA methylation—From epidemiological observations to experimental evidence
Source: Front Genet. 2022 Sep 13;13:993387. doi: 10.3389/fgene.2022.993387 (PMC9513252; doi:10.3389/fgene.2022.993387)
Supplement: Supplementary file 1 [file Table1.DOCX]

Supplementary Material

# Supplementary Figures and Tables

## Supplementary Figures


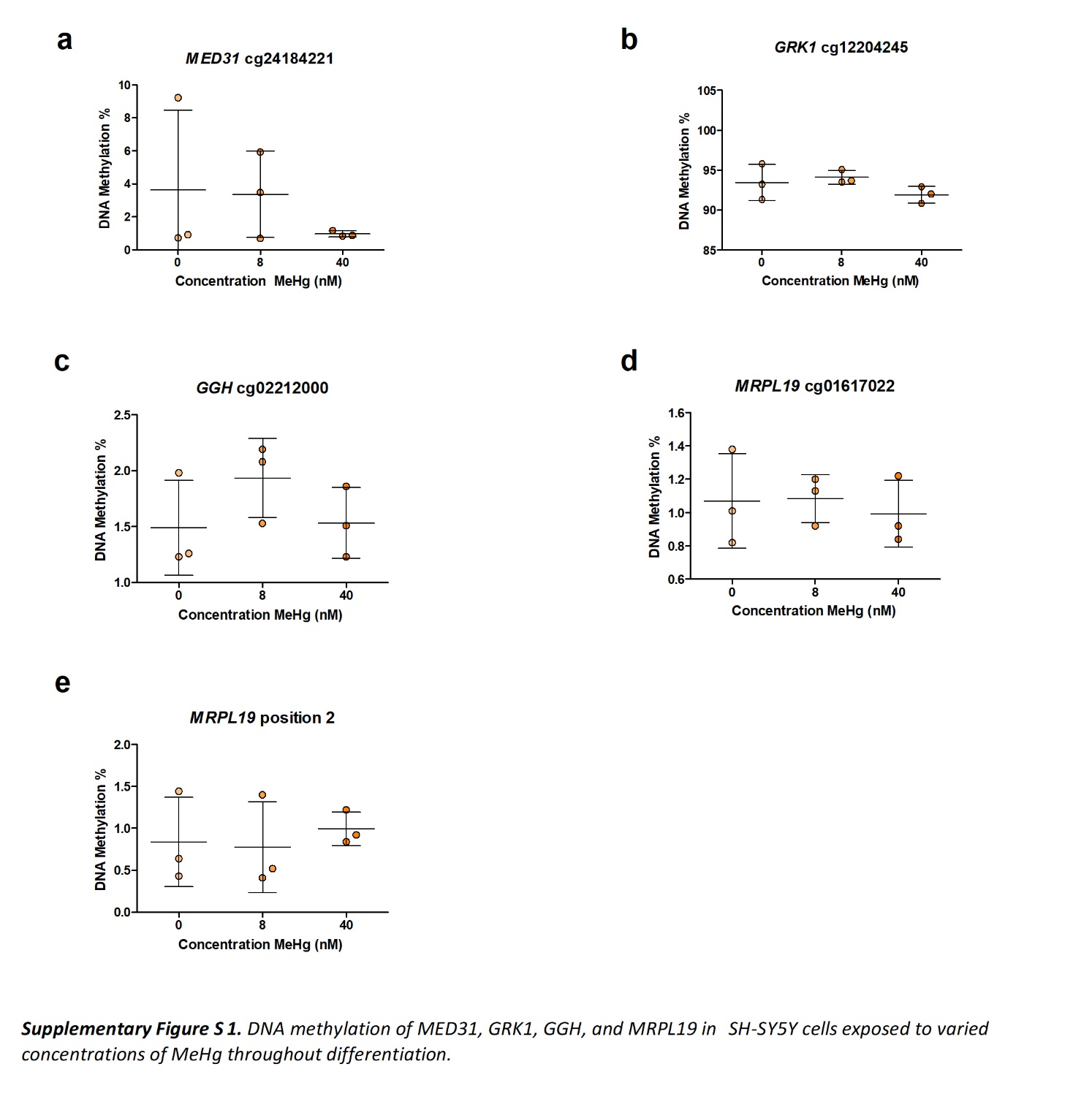


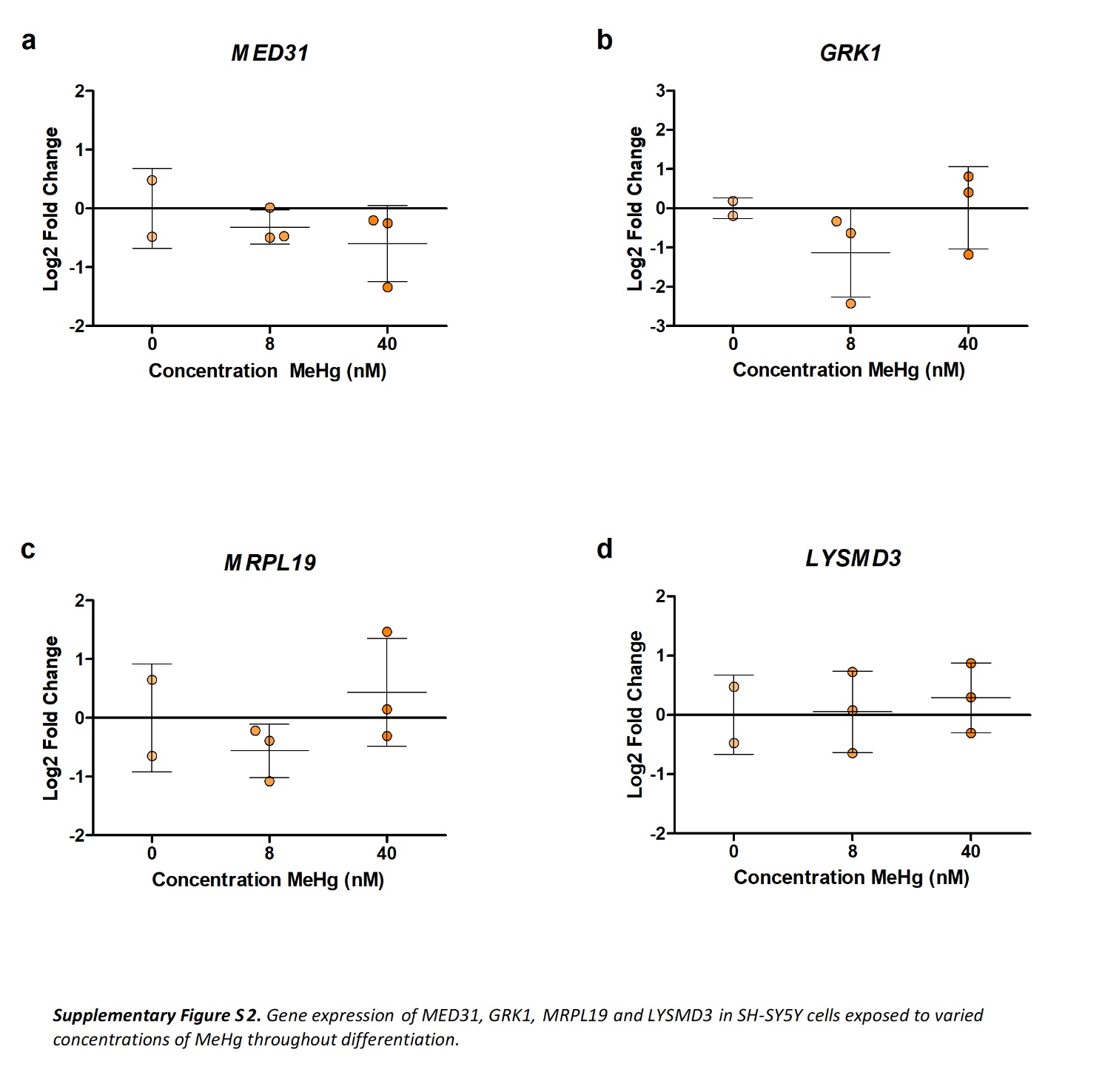


## Supplementary Tables

Supplementary table S1. Unique assay ID of the PrimePCR assays used for gene expression analyses

| Gene symbol | Unique Assay ID |
| --- | --- |
| *MRPL19* | qHsaCED0005648 |
| *GGH* | qHsaCID0014917 |
| *MED31* | qHsaCID0016698 |
| *LYSMD3* | qHsaCED0047148 |
| *GRK1* | qHsaCED0047789 |
| *TBP* | qHsaCID0007122 |
| *RPL19* | qHsaCED0005117 |
| *POLR2B* | qHsaCID0007449 |

Supplementary table S2. Assay conditions used for PCR and pyrosequencing of human and mouse selected sequences

| **Gene symbol** | **CpG cluster number (Illumina)** | **Position** | **Forward primer 5' - 3'** | **Reverse primer 5' - 3'** | **Sequencing primer 5' - 3'** | **Sequence analyzed** | **PCR conditions** | **Amplicon size** |
| --- | --- | --- | --- | --- | --- | --- | --- | --- |
| *MED31* | CpG1 (cg24184221) | chr17: 6,555,443 | TTTTTTGGTGGTTTTTGGGAAGTAGTATT | [Biotin]-ACCTCAACCCCAAAAACCAAACAAT | GTGAGTTGAGAGGAGGATT | TTAYGTGTYGGGAGGA | 95 °C for 15 min, 45 cycles of: 94 °C for 30 s, **57** °C for 30 s and 72 °C for 30 s; 72 °C for 10 min | 382 bp |
|  | CpG2 | chr17: 6,555,438 |  |  |  |  |  |  |
|  | CpG3 (cg15288800) | chr17: 6,555,742 | GTGAAAATAGTTTATTGGGTTAAAAGTAGG | [Biotin]-CCAACTACCAAATAAACCAATCAT | GTTGGTTGTGTTTAGAAAT | TGYGGTTTGG TTGTTTTTTA TATTGATT | 95 °C for 15 min, 45 cycles of: 94 °C for 30 s, **57** °C for 30 s and 72 °C for 30 s; 72 °C for 10 min | 157 bp |
| *MRPL19* | CpG1 (cg01617022) | chr2: 75,873,889 | AGGTTTATTGTAGTTTAGTGTTT | [Biotin]-AAAAAAACTTATCCATTTTATCCCATAAC | GTTAAGATTATAGTTTTTAGTAA | TTAGTGYGAY GAGGTTGTTG TTGTTTGTTA TTA | 95 °C for 15 min, 45 cycles of: 94 °C for 30 s, **57** °C for 30 s and 72 °C for 30 s; 72 °C for 10 min | 133 bp |
|  | CpG2 | chr2: 75,873,886 |  |  |  |  |  |  |
| *LYSMD3* | CpG1 (cg17282910) | chr5: 89,825,494 | GGGTTGGATTAGTTGATTTGGTTATTAAA | [Biotin]-ACCCAACCTCTACTTTAAACTAACCC | GGATTTTTTAGTTTTGGGTTGTT | TTTYGYGTTT TTTTT | 95 °C for 15 min, 45 cycles of: 94 °C for 30 s, **57** °C for 30 s and 72 °C for 30 s; 72 °C for 10 min | 355 bp |
| *GGH* | CpG1 (cg02212000) | chr8: 63,951,669 | TTGGGTTGGTTAGTTTAGTTTTG | [Biotin]-CCTTCCCTTTCAACTATTAC | TGTAAGGGTGATTGAAGTTTATA | TYGAYGTAATAGTTGAAAGGGAAGGA | 95 °C for 15 min, 45 cycles of: 94 °C for 30 s, **57** °C for 30 s and 72 °C for 30 s; 72 °C for 10 min *Contains 1µL of MgCl2 25 mM | 69 bp |
|  | CpG2 | chr8: 63,951,666 |  |  |  |  |  |  |
| *GRK1* | CpG1 (cg12204245) | chr13: 114,321,214 | TTTAAGTTTGTTGTTTTTTTGGGATGTG | [Biotin]-ATTTCTTCCCACTCCATCCTCT | GGGATGTGGGTTTGA | TTTGAYGGAGAGGATGGAGT | 95 °C for 15 min, 45 cycles of: 94 °C for 30 s, **58.5** °C for 30 s and 72 °C for 30 s; 72 °C for 10 min | 60 bp |

Human (GRCh37.p13 Primary Assembly)

Supplementary table S3. Transcription factor binding sites (TFBS) and transcription factor function. TFBS were predicted using University of California-Santa Cruz Genome Browser and the JASPAR2022 TFBS hg19 track.

| GENE SYMBOL | #CHROM | CHROMSTART | CHROMEND | NAME | SCORE | STRAND | FUNCTION |
| --- | --- | --- | --- | --- | --- | --- | --- |
| *GGH* | chr8 | 63951657 | 63951665 | MGA | 240 | + | repressor or activator |
| *GGH* | chr8 | 63951660 | 63951668 | ATF3 | 281 | + | repressor |
| *GGH* | chr8 | 63951660 | 63951668 | PAX2 | 238 | + | repressor or activator |
| *GGH* | chr8 | 63951660 | 63951670 | HIF1A | 272 | - | activator |
| *GGH* | chr8 | 63951662 | 63951670 | GMEB2 | 318 | + | activator |
| *GGH* | chr8 | 63951662 | 63951670 | GMEB2 | 373 | - | activator |
| *GGH* | chr8 | 63951671 | 63951678 | FOXO4 | 286 | - | activator |
| *MED31* | chr17 | 6555437 | 6555447 | HIF1A | 465 | - | activator |
| *MED31* | chr17 | 6555437 | 6555449 | HES5 | 444 | - | repressor |
| *MED31* | chr17 | 6555437 | 6555449 | HES7 | 457 | - | repressor |
| *MED31* | chr17 | 6555437 | 6555449 | MYC | 515 | - | activator |
| *MED31* | chr17 | 6555437 | 6555449 | MYCN | 442 | - | activator |
| *MED31* | chr17 | 6555437 | 6555450 | MXI1 | 470 | - | repressor |
| *MED31* | chr17 | 6555438 | 6555448 | BHLHE40 | 410 | - | repressor |
| *MED31* | chr17 | 6555438 | 6555448 | CLOCK | 407 | - | activator |
| *MED31* | chr17 | 6555438 | 6555448 | Hes1 | 414 | - | repressor |
| *MED31* | chr17 | 6555438 | 6555448 | HEY1 | 467 | - | repressor |
| *MED31* | chr17 | 6555438 | 6555448 | HEY2 | 424 | - | repressor |
| *MED31* | chr17 | 6555438 | 6555448 | MAX | 440 | - | repressor or activator |
| *MED31* | chr17 | 6555438 | 6555448 | MNT | 501 | - | repressor |
| *MED31* | chr17 | 6555438 | 6555448 | NPAS2 | 441 | - | activator |

Supplementary table S4. Spearman correlations between DNA methylation and gene expression observed in the analyzed regions.

| Gene | CpG number | Comparison | p-value (ANOVA) | Adjusted p-value (bonferroni) |
| --- | --- | --- | --- | --- |
| *MED31* | CpG1 (cg24184221) | 8-0 | 0.995 | 1 |
| *MED31* | CpG1 (cg24184221) | 40-0 | 0.562 | 1 |
| ***MED31*** | **CpG2** | **8-0** | **0.00175** | **0.0385** |
| *MED31* | CpG2 | 40-0 | 0.70243 | 1 |
| *MED31* | CpG3 (cg15288800) | 8-0 | 0.0988 | 1 |
| *MED31* | CpG3 (cg15288800) | 40-0 | 0.6456 | 1 |
| *MRPL19* | CpG1 (cg01617022) | 8-0 | 0.997 | 1 |
| *MRPL19* | CpG1 (cg01617022) | 40-0 | 0.903 | 1 |
| *MRPL19* | CpG2 | 8-0 | 0.985 | 1 |
| *MRPL19* | CpG2 | 40-0 | 0.905 | 1 |
| *LYSMD3* | CpG1 (cg17282910) | 8-0 | 0.85 | 1 |
| *LYSMD3* | CpG1 (cg17282910) | 40-0 | 0.974 | 1 |
| *LYSMD3* | CpG2 | 8-0 | 0.99 | 1 |
| *LYSMD3* | CpG2 | 40-0 | 0.777 | 1 |
| *GGH* | CpG1 (cg02212000) | 8-0 | 0.302 | 1 |
| *GGH* | CpG1 (cg02212000) | 40-0 | 0.989 | 1 |
| *GGH* | CpG2 | 8-0 | 0.583 | 1 |
| *GGH* | CpG2 | 40-0 | 0.721 | 1 |
| *GRK1* | CpG1 (cg12204245) | 8-0 | 0.857 | 1 |
| *GRK1* | CpG1 (cg12204245) | 40-0 | 0.431 | 1 |

Supplementary table S5. Spearman correlations between DNA methylation and gene expression observed in the analyzed regions.

| Gene | CpG number | Correlation with gene expression | |
| --- | --- | --- | --- |
|  |  | **r_S_** | **p-value** |
| *MED31* | CpG1 (cg24184221) | 0.24 | 0.58 |
| *MED31* | CpG2 | -0.048 | 0.93 |
| *MED31* | CpG3 (cg15288800) | -0.071 | 0.88 |
| *MRPL19* | CpG1 (cg01617022) | 0.54 | 0.17 |
| *MRPL19* | CpG2 | 0.5 | 0.22 |
| *LYSMD3* | CpG1 (cg17282910) | 0.41 | 0.32 |
| *LYSMD3* | CpG2 | 0.29 | 0.49 |
| *GGH* | CpG1 (cg02212000) | 0.3 | 0.47 |
| *GGH* | **CpG2** | **0.9** | **0.0046** |
| *GRK1* | CpG1 (cg12204245) | -0.5 | 0.22 |
